# Antibacterial activity of a berberine nanoformulation

**DOI:** 10.3762/bjnano.13.56

**Published:** 2022-07-11

**Authors:** Hue Thi Nguyen, Tuyet Nhung Pham, Anh-Tuan Le, Nguyen Thanh Thuy, Tran Quang Huy, Thuy Thi Thu Nguyen

**Affiliations:** 1 Phenikaa University Nano Institute (PHENA), Phenikaa University, Hanoi 12116, Vietnam; 2 National Institute of Hygiene and Epidemiology, Hanoi, Vietnam

**Keywords:** antibacterial activity, antisolvent precipitation (ASP), berberine nanoparticles (BBR NPs), glycerol, solubility

## Abstract

This study describes the preparation of berberine (BBR) in nanoformulation to enhance its solubility and increase its antibacterial effectiveness against hospital-acquired infections. BBR nanoparticles (BBR NPs) were formed by antisolvent precipitation (ASP) using glycerol as a safe organic solvent. UV–vis absorption spectra demonstrated that the solubility of BBR NPs was greatly enhanced compared to that of pure BBR. Glycerol played a role as a stabilizer for BBR NPs through the formation of hydrogen bonds between glycerol and BBR NPs. The prepared BBR NPs have a narrow size distribution with an average diameter of 156 nm at a concentration of 2.0 mg/mL, measured by dynamic light scattering. After nanoformulation, the concentration of BBR NPs could reach up to 5.0 mg/mL, which is much higher than the saturation concentration without treatment. Results show a strongly enhanced antibacterial activity of BBR NPs compared with that of pure BBR at the same concentration. The minimum bactericidal concentration of BBR NPs against methicillin-resistant *Staphylococcus aureus* and *Escherichia coli* O157:H7 was found to be 2.0 and 5.0 mg/mL, respectively. Transmission electron microscopy showed that BBR NPs surrounded the bacterial cells and severely damaged the cell walls. Therefore, BBR NPs prepared by ASP appear to be a potential candidate for the treatment of bacterial pathogens.

## Introduction

Phytochemicals are organic substances produced by plants with pharmacological and biological activity. Phytochemical-based medicines have become popular in the pharmaceutical market because of their diversity, availability, low cost, and little or no undesirable side effects [1]. Berberine (BBR) has been widely known as a phytochemical substance in treating diarrhea, digestive disorders, and gastroenteritis. It is the quaternary salt of the isoquinoline alkaloid extracted from many plants, such as *Berberis aristata*, *Berberis aquifolium*, *Berberis vulgaris*, *Coptis chinensis*, *Coptis japonica*, and *Hydrastis Canadensis* [2]. Many studies have shown the pharmacological actions of BBR including antimicrobial [3–4], antiviral [5–6], anti-inflammatory [7] and antiparasitic effects [8], and effects against hyperlipidemia [9], hypoglycemia [10] and cancer [11]. BBR has the ability to inhibit or kill many pathogenic bacteria, such as *Escherichia coli* [12], *Helicobacter pylori* [13], and *Staphylococcus aureus*, including methicillin-resistant strains and coagulase-negative strains [4,14]. The antibacterial activity of BBR against *Streptococcus agalactiae* was explained by BBR molecules damaging the structure of the bacterial cell membrane and inhibiting the synthesis of proteins and DNA [15]. Chu et al. [16] reported that BBR showed no antibacterial activity against methicillin-resistant *Staphylococcus aureus* (MRSA) in the range of concentrations from 1 to 64 µg/mL. However, inhibition of MRSA biofilm formation was remarkable at concentrations greater than 8 µg/mL through affecting the aggregation of phenol-soluble modulins into amyloid fibrils. This result suggests that BBR may be a therapeutic agent against microbial-generated amyloid-involved diseases. BBR is also a phytochemical exhibiting a strong antiviral activity against different viruses, including human cytomegalovirus [17], enterovirus 71 [18], H_1_N_1_ influenza virus [19], and human immunodeficiency virus (HIV) [6]. BBR has been shown to inhibit viral replication by specific targets and increase the host immune response for viral clearance [20]. Recent studies have shown evidence that BBR and its derivatives can also fight severe acute respiratory syndrome coronavirus 2 (SARS-CoV-2), which is a current concern worldwide [21–23].

The water solubility of phytochemicals plays an important role in the effectiveness of disease treatment. A poor water solubility of a drug leads to low drug absorption. Thus, a sufficient drug concentration in plasma is not achieved, and a high therapeutic effect is not reached. As a result, high dose requirements and more adverse side effects limit the development of phytochemicals in the pharmaceutical industry. BBR belongs to the class-III drugs in the biopharmaceutical classification system, indicating that BBR is a lipophilic compound that has poor absorption and low bioavailability [24]. To improve the effectiveness of BBR, many approaches have been proposed, including synergistic effects with other drugs [3], particle size reduction [25], and encapsulation in nanoscale delivery systems [11]. Nanoscale BBR crystals can be formed using top-down technologies (ball mills, high-pressure homogenizers, microfluidic technology, and spray drying) or bottom-up technologies (evaporative precipitation of nanosuspension (EPN) and antisolvent precipitation (ASP)). Although top-down technologies have a high yield, they are energy-consuming and the obtained crystal size is inhomogeneous [26–27]. The ASP technique can modify crystal formation and particle size distributions [5]. Sahibzada et al. [26] reported that BBR nanoparticles produced by EPN and ASP techniques have notably increased solubility and dissolution rate due to their semicrystalline form. The small size of the nanoparticles can be obtained by adjusting the drug concentration, flow rate, stirring rate, and antisolvent volume in EPN and ASP methods [28].

Many types of nanoformulations, such as polymer-, lipid-, dendrimer-, graphene-, gold-, or silver-based nanoparticles, have been used for the delivery of BBR [29–31]. Yu et al. [29] prepared poly(ethylene glycol)–lipid–poly(lactic-*co*-glycolic acid) nanoparticles loaded with BBR to improve the oral delivery efficiency of BBR. This nanoformulation increased the oral relative bioavailability to approximately 343% compared to that of the original BBR. Although nanoformulations have emerged as an effective approach to improve the bioavailability of BBR, several drawbacks should be addressed as follows: (1) Almost all drug-carrying nanomaterials have high cost, (2) the drug concentration of the encapsulated form is usually low, and (3) the toxicity risk of nanomaterials must be strictly controlled [32–34]. Most nanoformulations have relatively low drug loading (typically a few weight percent), and it remains a challenge to increase drug loading. Besides, the high cost and the complexity of nanoformulation production narrow the accessibility [32]. Regarding dendrimers, micelles, and polymeric nanoparticles, there are also issues with long-term stability, low drug loading efficiency, and batch-to-batch variations [33]. Also, non-biodegradability and long-term toxicity are limitations of inorganic nanocarriers [34].

The purpose of this study was to fabricate BBR nanoparticles (BBR NPs) without using any nanocarriers, thus reducing production cost, increasing the drug concentration, and eliminating toxic ingredients in the formulation. The BBR NPs were formed in order to improve the solubility and, in turn, enhance the antibacterial activity. Herein, the BBR NPs were prepared using a modified ASP method including sonication steps. Instead of the solvents utilized in previous reports [25–26], glycerol, a safe substance in pharmaceutical applications, was used to dissolve BBR in this study. Sonication provided mechanical energy to improve the dispersion of the nanosized BBR crystals. Homogeneous BBR NPs were produced with a size of 156 nm under a certain sonication conditions. The solubility of BBR was significantly enhanced. The concentration of BBR NPs in aqueous solution (3.0 mg/mL) was higher than the saturation concentration of the precursor BBR (lower than 2.0 mg/mL). A notable increase in the antibacterial activity of BBR NPs against representative Gram-positive (MRSA) and Gram-negative (*Escherichia coli* O157:H7) bacteria causing hospital-acquired infections was achieved in comparison with the precursor BBR at the same concentration. In addition, the interaction of BBR NPs with bacteria was also investigated in this study.

## Results and Discussion

### Chemical characterization of the BBR NPs

Solvent molecules strongly affect the formation of crystalline structures and the stabilization of crystal lattices [35]. To the best of our knowledge, in most published papers regarding the synthesis of BBR NPs via the ASP method ethanol or methanol were used as solvents. This study is the first to report that glycerol can serve as an effective green solvent for BBR NP formation. Being non-toxic, renewable, and biodegradable, and having a suitable dielectric constant to dissolve various compounds such as BBR, glycerol is a promising candidate to replace toxic organic solvents. The effect of glycerol on the physicochemical properties of BBR NPs was investigated by UV–vis absorption and FTIR spectroscopy.

**Figure 1:**
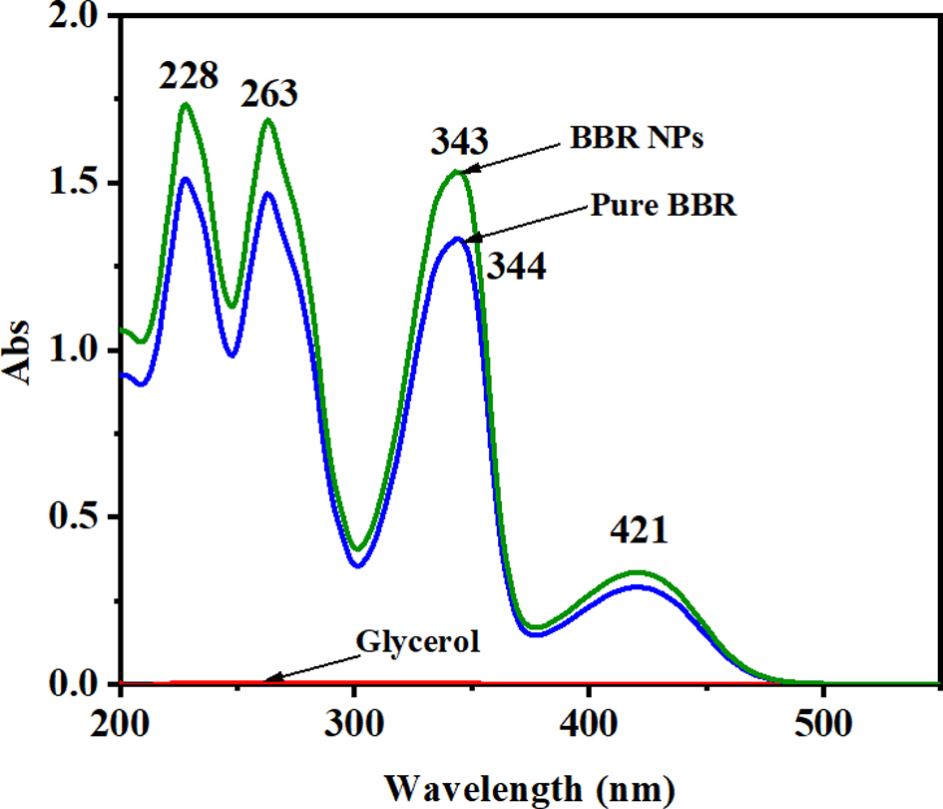
UV–vis spectra of BBR and BBR NPs in distilled water at a concentration of 2.0 mg/mL.

The UV–vis absorption spectra of pure BBR and BBR NP solutions in distilled water at the same concentration are shown in Figure 1. Glycerol has no absorption band in the UV–vis spectral region because it does not possess a conjugated structure. There are peaks of three strong bands at 228, 263, and 344 nm in the UV–vis spectra of pure BBR and BBR NP solutions. These peaks characterize the π–π* transition in the BBR molecular structure. One weak band is centered at 421 nm, attributed to the n–π* transition band [36]. No change in peak positions was found. However, the peak intensities of BBR NP solution were higher than those of pure BBR solution at a concentration of 2.0 mg/mL. This finding may be because the saturation concentration of pure BBR is lower than 2.0 mg/mL, whereas the solubility of BBR NPs was significantly enhanced.

**Figure 2:**
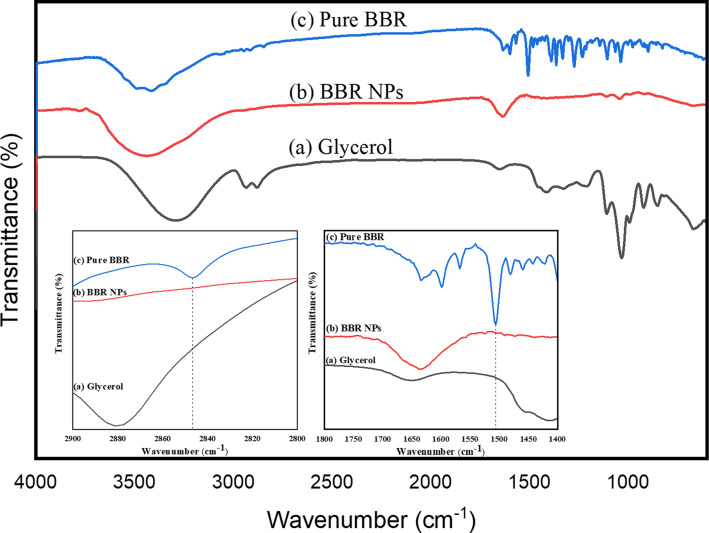
FTIR spectra of (a) glycerol, (b) pure BBR, and (c) BBR NPs at a concentration of 2.0 mg/mL.

The chemical characteristics of BBR and BBR NPs were analyzed through FTIR spectroscopy (Figure 2). In the FTIR spectrum of glycerol (Figure 2a), the absorption band appearing at 3287 cm^−1^ is characteristic for stretching vibrations of the –OH group. The two bands with maxima at 2934 and 2880 cm^−1^ are ascribed to symmetrical and asymmetrical –CH_2_ vibrations. The band at 1032 cm^−1^ is attributed to C–H deformation vibrations and C–C stretching vibrations. In the FTIR spectrum of pure BBR (Figure 2c), an intense broad band at 3414 cm^−1^ appeared because of O–H stretching vibrations of moisture in the samples. A weak absorption band at 2844 cm^−1^ was assigned to the stretching vibrations of the methoxy group (–O–CH_3_) [37]. In addition, the peak at 1633 cm^−1^ characterized the C=N^+^ double bond in the molecular structure of BBR. Characteristic peaks found at 1567 and 1506 cm^−1^ were attributed to the vibrations of the C=C bonds in the aromatic ring and the furyl group, respectively. However, in the FTIR spectrum of BBR NPs (Figure 2b), the characteristic peaks at 2844 and 1506 cm^−1^ did not appear (zoomed insets of Figure 2). This finding can be explained by the formation of hydrogen bonds between the oxygen-containing groups (methoxy and furyl groups) of BBR and the –OH group of glycerol in water [38].

### Morphology and size distribution of BBR NPs

**Figure 3:**
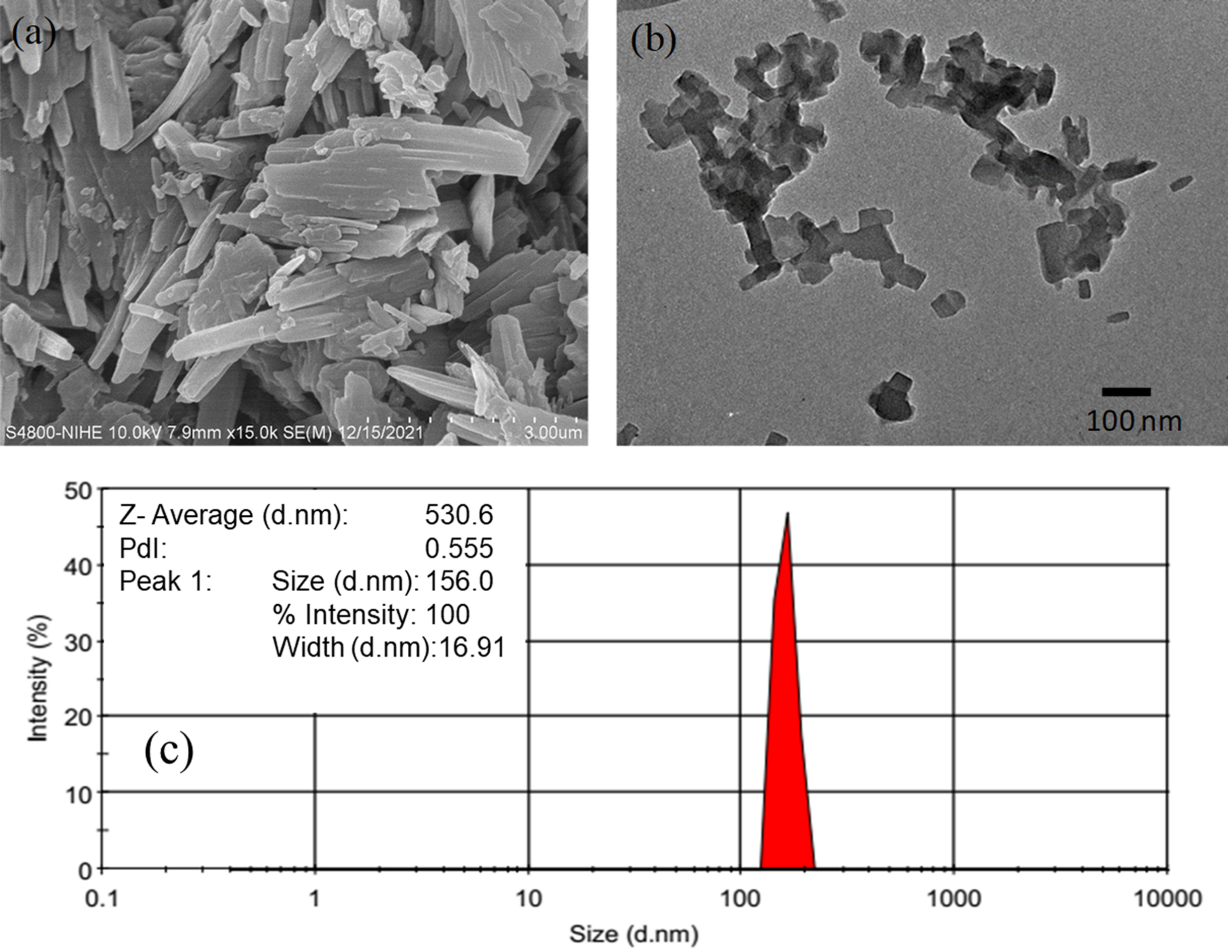
Morphology of (a) pure BBR and (b) BBR NPs and (c) DLS measurement of BBR NPs.

The SEM image (Figure 3a) shows that pure BBR forms tightly agglomerated rods with rectangular cross section and different sizes in the micrometer range. After the antisolvent precipitation process, the size of BBR NPs was expected to be at the nanoscale. TEM observation shows that the BBR NPs had a uniform rectangular shape with sizes lower than 100 nm (Figure 3b). It also reveals a good dispersion of BBR NPs. The mean size of the particles measured by DLS was 156 nm for BBR NPs at a concentration of 2.0 mg/mL. The particle size measured by DLS was larger than the size obtained from TEM analysis because this dynamic measurement is affected by the concentration of the solution, surface properties of the particles, polydispersity, and the agglomeration of particles during DLS analysis [39]. The BBR NPs prepared at a concentration of 2.0 mg/mL showed a narrow size distribution by intensity (Figure 3c). The *z*-average diameter of BBR NPs was 530.6 nm (Supporting Information File 1). In addition, its PdI value reached 0.555, indicating a good stability of these BBR NP solutions. This can be explained by the narrow distribution of particle sizes, indicated by the low PDI value, resulting in a sufficiently large repulsive interaction between particles to form a stable dispersion [40]. Glycerol is a solvent that possesses many hydroxy groups. The hydrogen atoms of these hydroxy groups and the oxygen atoms of methoxy and furyl groups of BBR form hydrogen bonds, which can effectively avoid the aggregation of BBR NPs. Therefore, glycerol acts as a stabilizer to improve the distribution and stability of BBR NPs in water.

### Antibacterial activity

**Figure 4:**
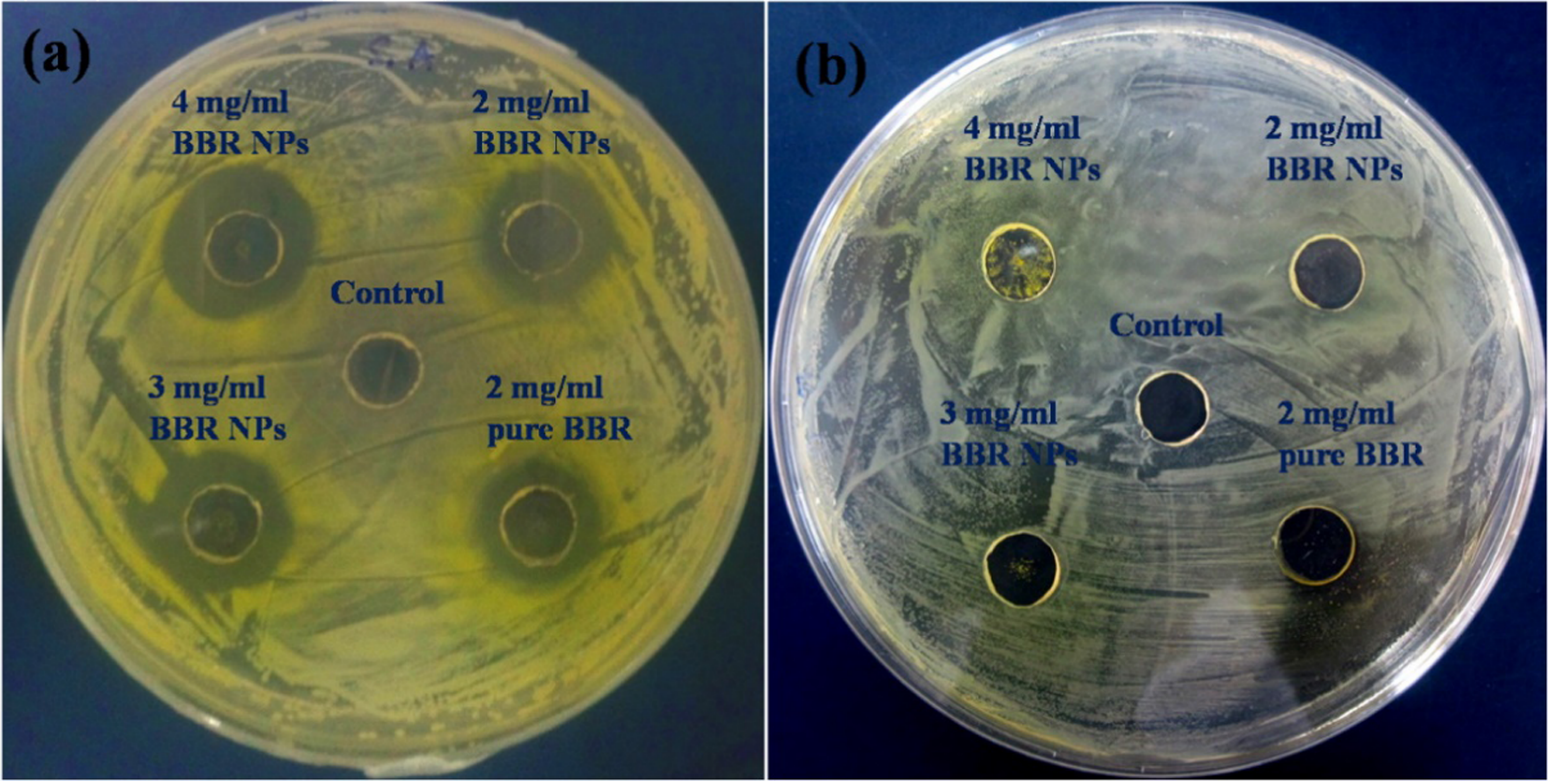
Antibacterial activity of negative control, pure BBR at the saturation concentration of 2.0 mg/mL, and BBR NPs at concentrations of 2.0, 3.0, and 4.0 mg/mL against (a) MRSA and (b) *E. coli* O157:H7 determined by the modified disk diffusion method.

The antibacterial activity of pure BBR and BBR NPs prepared at different concentrations against MRSA and *E. coli* O157:H7 was compared in vitro using the modified disk diffusion method. Figure 4 and Table 1 show the inhibitory zones of pure BBR (at the saturation concentration of 2.0 mg/mL) and BBR NPs (2.0, 3.0, and 4.0 mg/mL) against MRSA and *E. coli* O157:H7. An inhibitory zone with a diameter of 15 mm was found for pure BBR against MRSA at a concentration of 2.0 mg/mL (Figure 4a). At the same concentration, BBR NPs gave a higher diameter of the inhibitory zone (16 mm). When the concentration of BBR NPs was increased from 2.0 to 4.0 mg/mL, the diameter of the inhibitory zone significantly increased from 16 to 19 mm (Table 1). This finding confirmed that BBR and BBR NPs were highly effective to inhibit the growth of MRSA at a concentration of at least 2.0 mg/mL. Higher concentrations of BBR could be obtained due to the nanoformulation. Thus, the antibacterial activity could be enhanced. In contrast, determining the inhibition zones against *E. coli* O157:H7 at different concentrations was very difficult (Figure 4b). Therefore, this method is insufficiently reliable to evaluate the antibacterial activity of BBR and BBR NPs against *E. coli* O157:H7. However, our study indicated that the antibacterial activity of BBR is more effective against Gram-positive bacteria (MRSA) than against Gram-negative bacteria (*E. coli* O157:H7). The studies showed that BBR has weaker inhibitory ability against Gram-positive bacterial strains than in published previously results [41–42].

**Table 1:** Diameter of the inhibitory zone against MRSA of pure BBR and BBR NPs at different concentrations.

Sample	Pure BBR 2.0 mg/mL	BBR NPs 2.0 mg/mL	BBR NPs 3.0 mg/mL	BBR NPs 4.0 mg/mL

diameter of the inhibitory zone (mm)	15	16	17	19

**Figure 5:**
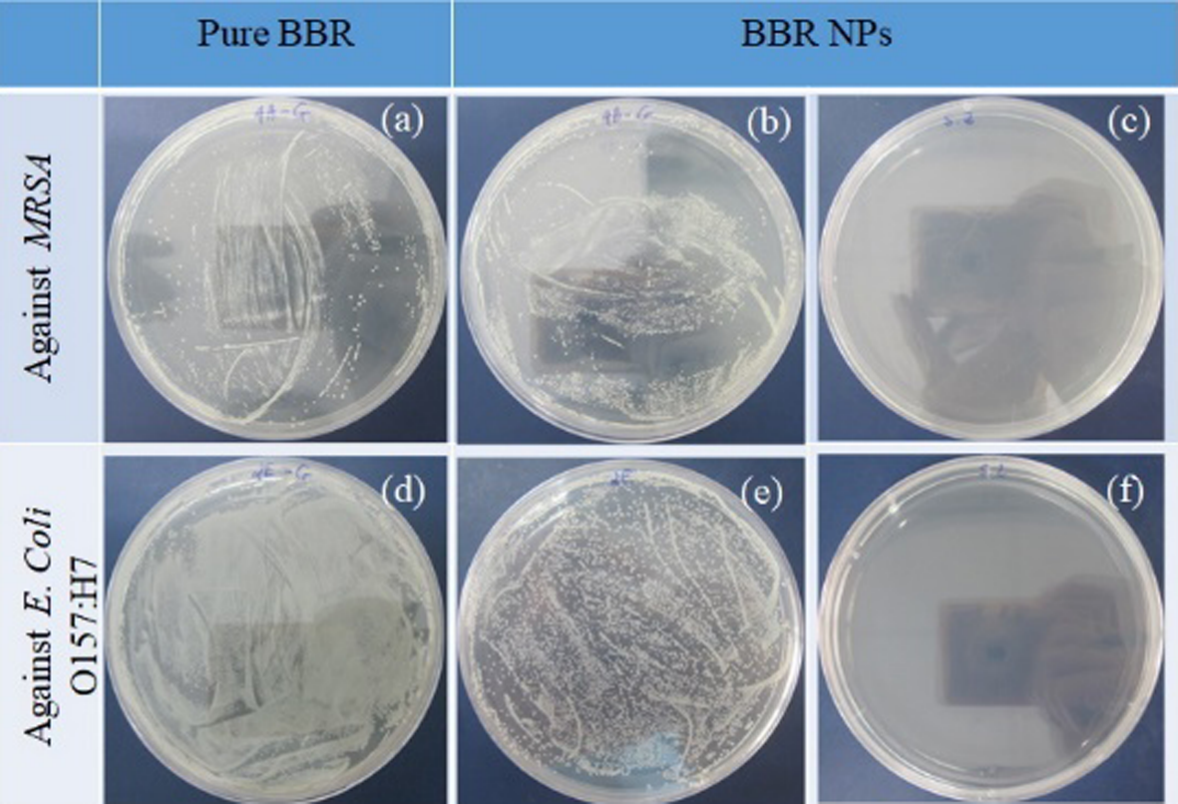
Agar plates inoculated with solutions incubated with pure BBR at the saturation concentration of 2.0 mg/mL and (a) MRSA and (d) *E. coli* O157:H7. Agar plates inoculated with solutions incubated with BBR NPs at concentrations of (b) 1.0 mg/mL (MRSA), (c) 2.0 mg/mL (MRSA), (e) 4.0 mg/mL (*E. coli* O157:H7), and (f) 5.0 mg/mL (*E. coli* O157:H7).

For the inhibitory concentrations, the results were determined using 48-well plates (see the sample layout below in Figure 8, Experimental section). Growth of bacterial strains in the wells of rows 5–7 in all plates was observed, given the turbidity that formed compared with the wells of the positive controls. However, confirming whether the agents could inhibit the growth of bacterial strains in rows 1–6 was difficult due to the interference of the yellow color of BBR and BBR NPs at high concentrations. Therefore, the solution of these rows was inoculated onto agar surface in Petri dishes for determination of the minimum bactericidal concentration (MBC, Figure 5). The results showed that the MBC values of BBR NPs against MRSA and *E. coli* O157:H7 were 2.0 and 5.0 mg/mL, respectively. No MBC was found for pure BBR up to the saturation concentration of 2.0 mg/mL.

**Figure 6:**
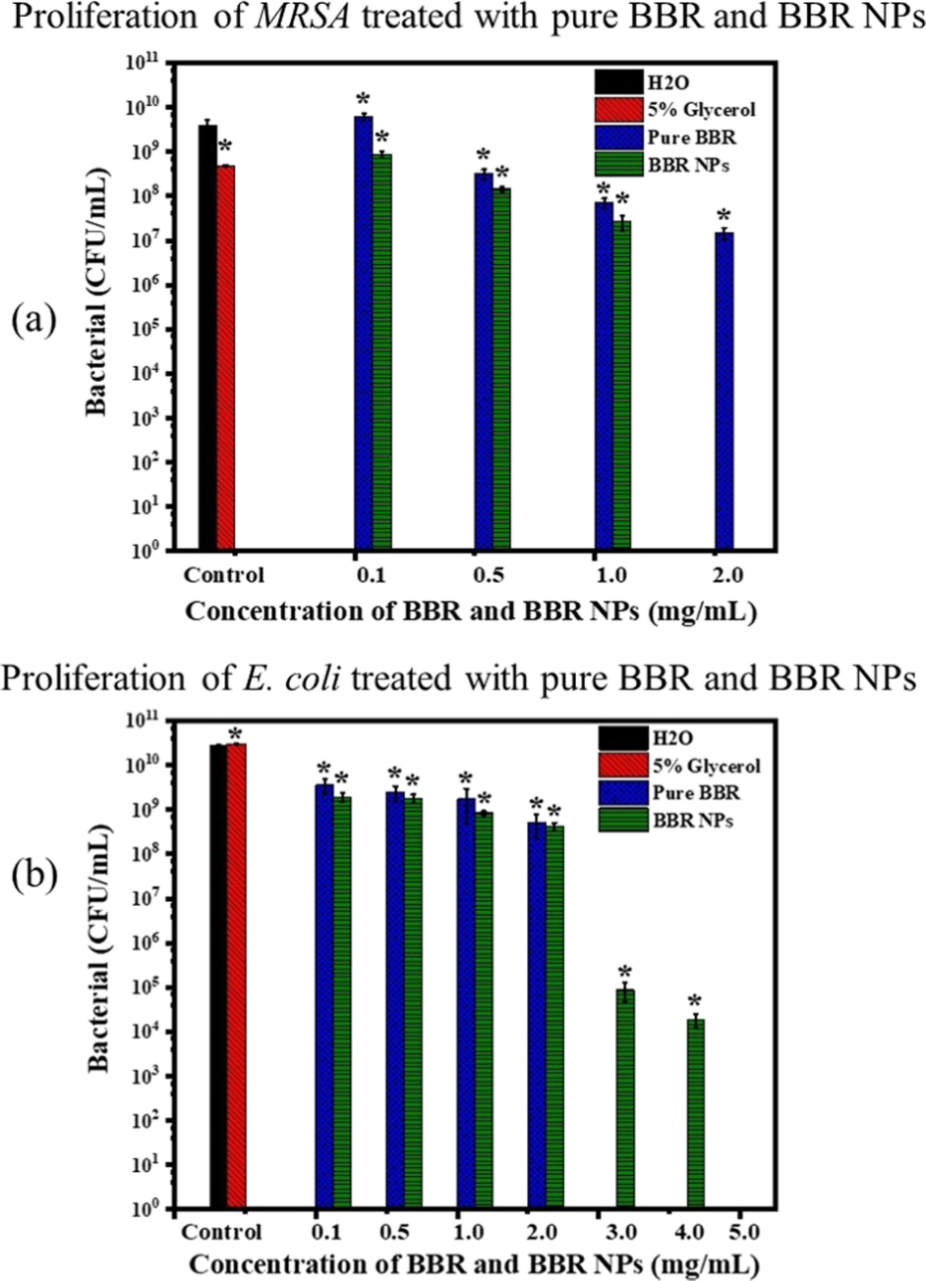
Antibacterial efficacy of negative control, pure BBR (2.0 mg/mL), and BBR NPs at concentrations of 2.0, 3.0, 4.0, and 5.0 mg/mL against (a) MRSA and (b*) E. coli* O157:H7 determined by the colony counting method. Bars show mean value ± standard deviation (*n* = 3) and the asterisks indicate a *p*-value smaller than 0.05.

Figure 6 shows the proliferation of MRSA and *E. coli* O157:H7 in nutrient broth after treatment with pure BBR and BBR NPs at different concentrations between 0.1 and 5.0 mg/mL. The results were obtained by colony counting on agar discs after inoculating with the diluted nutrient broth in the tested 48-well plates. Figure 6a shows that pure BBR did not exhibit inhibitory activity against MRSA at a concentration of 0.1 mg/mL. However, the inhibitory activity gradually increased when the concentration of pure BBR increased from 0.5 mg/mL to the saturation concentration of BBR (2.0 mg/mL). The comparison between pure BBR and BBR NPs indicates that the antibacterial activity of BBR NPs was slightly higher in the concentration range of 0.1–1.0 mg/mL. Notably, proliferation of MRSA was not observed after treatment with BBR NPs at a concentration of 2.0 mg/mL. BBR NPs at a concentration of 2.0 mg/mL and higher exhibited a strong antibacterial effect against MRSA.

Pure BBR and BBR NPs show low inhibitory activity at concentrations between 0.1 and 2.0 mg/mL against *E. coli* O157:H7 (Figure 6b). The proliferation of *E. coli* O157:H7 after treatment with pure BBR and BBR NPs at the same concentrations was insignificantly different. The reduction in the proliferation of *E. coli* O157:H7 was clearly observed after BBR NP treatment at concentrations of 3.0 and 4.0 mg/mL. Meanwhile, after applying the MBC of 5.0 mg/mL, no surviving bacteria were found. This finding proved that BBR NPs had inhibitory ability against *E. coli* O157:H7 at high concentrations. In our study, 5 wt % glycerol showed low inhibitory activity against MRSA, and no inhibitory activity against *E. coli* O157:H7.

Determining the antibacterial activity of BBR NPs by colony counting was more reliable than by the modified disk diffusion method. This finding can be explained by the low diffusion of BBR NPs on the agar surface. BBR NPs interacted more with bacteria in the nutrient broth for the colony counting method.

BBR NPs with a high surface-to-volume ratio facilitate interaction and absorption onto the bacterial cell membrane given the benefit of nanoscale size. According to previous studies, BBR can penetrate the phospholipid bilayers and then accumulate in the MRSA cell membrane, in which unsaturated fatty acids are the target of BBR-induced reactive oxygen species, leading to cell integrity deterioration [43].

### Interaction of BBR NPs with bacteria

**Figure 7:**
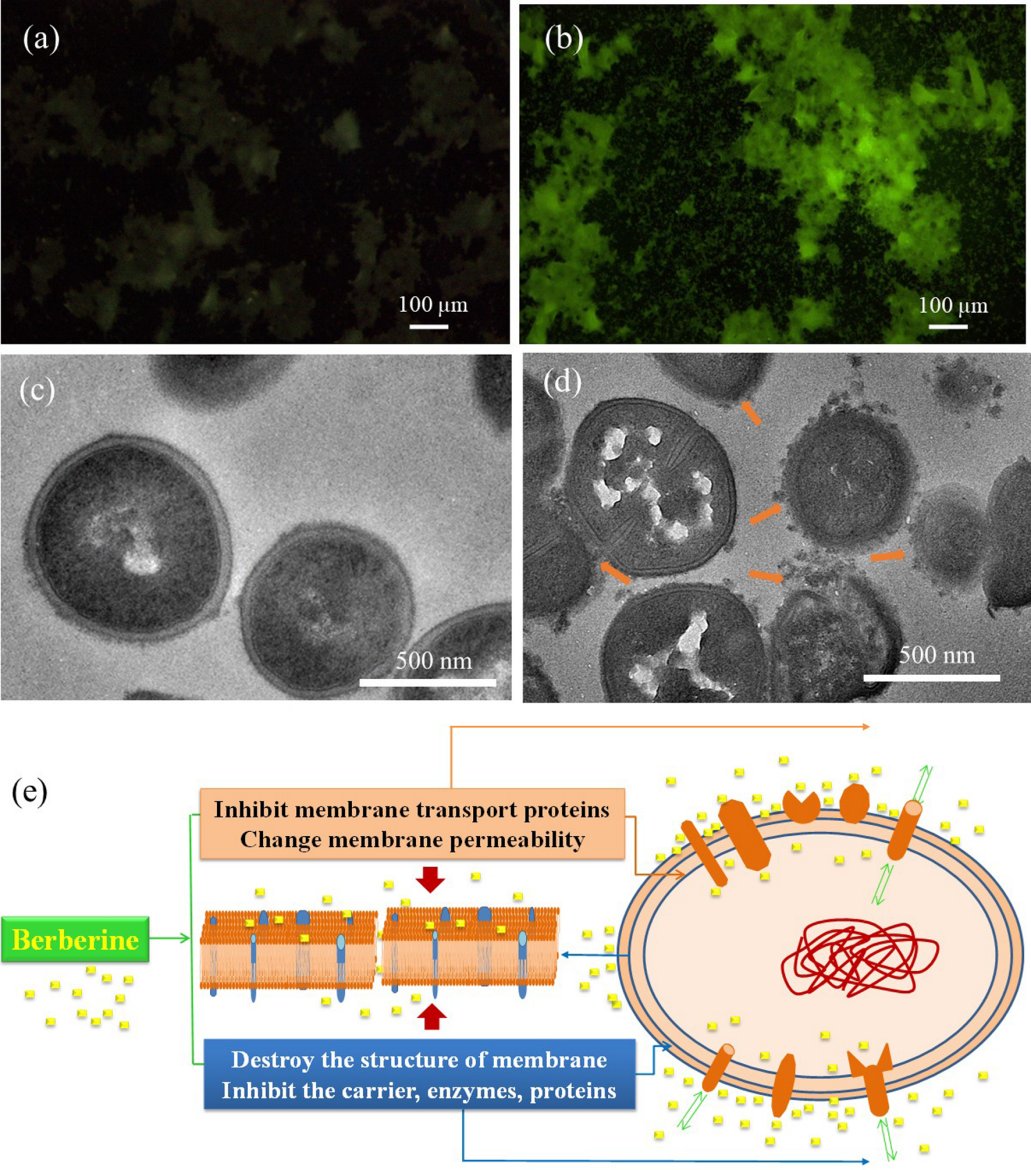
Fluorescence stereomicroscopy images of MRSA (a) without and (b) after treatment with BBR NPs; cross-sectional TEM images of MRSA cells (c) without and (d) after treatment with BBR NPs; (e) proposed mechanism of BBR NPs against MRSA cells.

In this study, bacterial strains of MRSA and *E. coli* O157:H7 were tested with 2 mg/mL of BBR NPs to investigate the interaction between these nanoparticles and bacterial cells. We used ultrathin sectioning for TEM observations. We did not find any difference in ultrastructural changes of *E.coli* O157:H7 cells treated with and without BBR NPs, whereas significant differences were found in MRSA cells after treatment with BBR NPs. This is consistent with results obtained using the disk diffusion method. MRSA is a Gram-positive bacterium that has a thick and smooth peptidoglycan layer in the cell wall. Furthermore, the above results also revealed that the antibacterial activity of BBR NPs against MRSA is higher than that against *E. coli* O157:H7. Figure 7a shows an unclear fluorescence stereomicroscopy image of MRSA. The image is unclear because the bacterial cells are not self-fluorescent under excitation. In contrast, after incubation with BBR NPs, the BBR molecules adhered to the bacterial cell walls exhibited fluorescence (Figure 7b) upon UV excitation [44]. In our experiment, the samples used for observation under the fluorescence microscope were all centrifuged and washed three times in bi-distilled water. However, due to the limited resolution of the stereomicroscope, the interaction of BBR NPs and bacterial cell walls could not be clearly observed. Therefore, the samples were processed by ultrathin sectioning to observe their ultrastructure under the transmission electron microscope. Figure 7c shows a cross-sectional image of MRSA cells under the impact of BBR NPs. The image shows that BBR NPs surround the bacterial wall and adhere to it. At the binding sites, the outer layer of the bacterial wall becomes rough and damaged (arrow). At sites without influence of BBR NPs, the bacterial cell wall remained intact. This result confirmed that the BBR NPs have approached and adhered to the bacteria, damaging the cell wall, enabling smaller particles of BBR to go deep into the cells and to damage the cells (Figure 7d). All these processes can be responsible for the death of bacterial cells [45]. Using TEM, it is very difficult to see the interaction between bacterial cells and BBR molecules at the ultrastructural level. However, it can be seen that, where the BBR NPs adhere to the bacterial, their cell wall is not intact.

## Conclusion

BBR has been successfully nanoformulated with high homogeneity and concentration using the ASP method. Glycerol is not only a solvent for BBR in the ASP process but also a stabilizer for BBR NPs due to the hydrogen bonds between glycerol and BBR molecules. The solubility of BBR NPs was significantly enhanced. Thus, the concentration of BBR NPs could reach up to 5.0 mg/mL, which was much higher than the saturation concentration of pure BBR. The diameter of the BBR NPs measured with dynamic light scattering was 156 nm at a concentration of 2.0 mg/mL. BBR NPs at a concentration of 2.0 mg/mL could completely kill MRSA at a bacterial concentration of about 5 × 10^5^ CFU/mL, while pure BBR only showed inhibitory activity. The MBC of BBR NPs against *E. coli* O157:H7 was 5.0 mg/mL. The great improvement in antibacterial activity indicated that the proposed BBR NPs are a promising bactericidal drug in clinical practice, especially against hospital-acquired infections.

## Experimental

### Materials

BBR chloride was purchased from Sigma-Aldrich (pharmaceutical primary standard). Glycerol was provided by Fisher Scientific (purity > 99%). Nutrient broth and nutrient agar were obtained from Titan Biotech, India. Bi-distilled water from a Milli-Q system (18.2 MΩ·cm at 25 °C) was used. All other chemicals were of analytical grade and used without further purification.

Two bacterial strains isolated from clinical samples, namely, methicillin-resistant *Staphylococcus aureus* (MRSA) and *Escherichia coli* O157:H7 (*E. coli* O157:H7) were provided by the National Institute of Hygiene and Epidemiology, Vietnam.

### Methods

#### Preparation of BBR NPs and saturated BBR solution

BBR NPs were prepared through the ASP method and subsequently sonicated in a bath sonicator. A total of 100 mg of BBR was dissolved in 1 mL of glycerol at 37 °C to obtain a clear, orange solution. Then, the freshly prepared solution was dripped at a rate of 1 mL/min into a beaker containing 25 mL bi-distilled water as an antisolvent under stirring at 1000 rpm. While the volume of the antisolvent remained constant, that of the dissolved BBR was varied to obtain the solutions with different concentrations of BBR (0.1, 0.5, 1.0, 2.0, 3.0, 4.0, and 5.0 mg/mL). Finally, all samples were placed in a bath sonicator for 5 min and stored at room temperature for further analyses.

Saturated BBR solution was produced by adding 2.0 mg raw BBR in a 2.0 mL Eppendorf tube containing 1.0 mL of distilled water. The tube was placed in an ultrasonic bath for 30 min at room temperature. Then, the BBR solution was centrifuged at a speed of 5000 rpm for 5 min. The supernatant liquid was withdrawn as saturated BBR solution.

#### Physicochemical properties of BBR NPs

UV–vis absorption spectra of the aqueous solutions of pure BBR and BBR NPs having the same concentration were measured in the wavelength range of 200–550 nm using a UV–vis spectrophotometer (6850 UV/Vis, Jenway). The chemical bonding characteristics of pure BBR powder and BBR NPs solution were determined using Fourier-transform infrared spectroscopy (FTIR, NEXUS 670 from Nicolet). The FTIR analysis was conducted in transmission mode in the wavenumber range of 400 to 4000 cm^−1^. Size and shape of BBR NPs were investigated by scanning electron microscopy (SEM, S-4800, Hitachi) and transmission electron microscopy (TEM, JEM1010, JEOL). The dynamic average size distribution and the stability of the prepared BBR NPs in aqueous solution were evaluated by dynamic light scattering (DLS) using a Zetasizer Nano ZS, Malvern Instruments.

#### Antibacterial activity of BBR NPs

The antibacterial activity of the pure BBR at saturation concentration and BBR NPs at different concentrations of 0.5 to 5.0 mg/mL was examined against two representative bacteria causing hospital-acquired infections including a Gram-positive strain (MRSA) and a Gram-negative strain (*E. coli* O157:H7) in vitro using the modified disk diffusion method, determination of the minimum bacterial concentration, and colony counting. In the modified disk diffusion method, sterile plastic Petri dishes (90 mm) were prepared with 20 mL of nutrient agar each, and then 100 µL of the bacterial suspension at a concentration of 10^7^ CFU/mL was dripped onto the agar surface and spread evenly with a glass triangle stick. The agar holes were made with a diameter of 8 mm. Then, 100 µL of samples of saturation concentration of BBR (2.0 mg/mL) and different concentrations of BBR NPs (2.0, 3.0, and 4.0 mg/mL) were gently loaded into the holes. The control hole was added with bi-distilled water. Subsequently, the dishes were covered and placed on the clean bench for 3 h at room temperature for letting the solution diffusing out of the holes. Finally, the dishes were incubated at 37 °C for 24 h. The diameter of the inhibitory zones was recognized and measured surrounding the holes.

**Figure 8:**
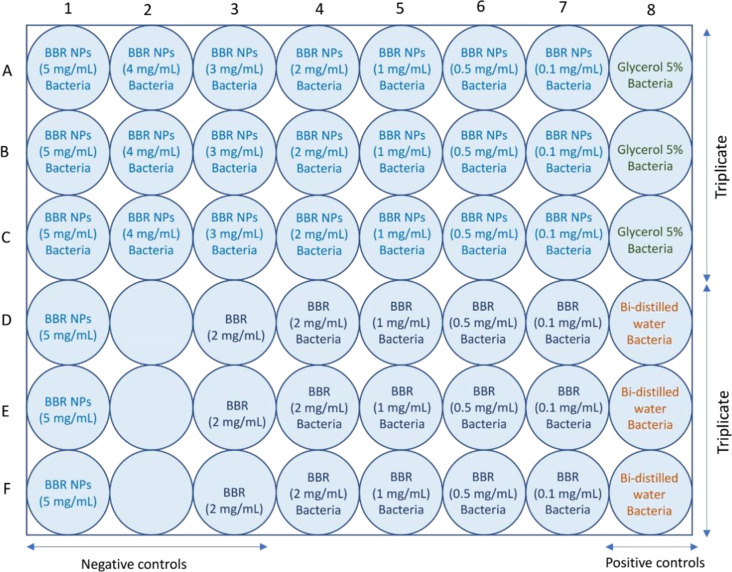
Experimental design for testing the inhibitory concentrations of BBR NPs and BBR against bacterial pathogens.

For the determination of the inhibitory concentration, BBR and BBR NPs were diluted and tested with bacterial strains in a sterilized 48-well plate (six rows lettered from A to F, and eight columns numbered from 1 to 8). First, all wells were prepared with 180 µL of the nutrient broth. In the case of BBR NPs, in rows A–C, columns 1–7 were added with 200 µL of the sample per well, corresponding to concentrations of 5.0, 4.0, 3.0, 2.0, 1.0, 0.5, and 0.1 mg/mL. Column 8 was added with 200 µL of 5.0% glycerol/water per well and served as the positive control. In the case of BBR, rows D–F, columns 4–7 were added with 200 µL of the sample corresponding to concentrations of 2.0, 1.0, 0.5, and 0.1 mg/mL. Column 8 was added with 200 µL of bi-distilled water per well and served as the positive control. Subsequently, 20 µL of bacterial strains at a concentration of 10^7^ CFU/mL were added to all wells except the wells in columns 1–3 of rows D–F, which served as negative controls. The plate was incubated at 37 °C for 24 h. The initial inhibitory concentration was determined via the turbidity of the wells with the samples compared with those of the negative and positive controls. The sample layout is presented in Figure 8. The antibacterial activity of BBR and BBR NPs was confirmed again via determining the minimum bacterial concentration (MBC).

For MBC determination, 20 µL of the inoculated solutions in the wells was diluted into 80 µL of physiological saline, spread onto the agar surface in Petri dishes, and then incubated at 37 °C for 24 h. The remaining inoculated solutions in the 48-well plate were maintained in the fridge at 2–4 °C for further investigation. After MBC determination, the inoculated solutions in the wells were continuously diluted tenfold in physiological saline from the initial concentration. Subsequently, these solutions were spread onto the agar surface in Petri dishes and incubated at 37 °C for colony counting. After 24 h, the number of bacterial colonies was counted on the agar surface. The difference in bacterial colonies corresponds to the inoculated solution in wells tested with BBR or BBR NPs, and the same diluted concentration could confirm exactly the antibacterial effectiveness of BBR before and after nanoformulation.

#### Observation of the bacteria–BBR NP interaction

Their interaction between BBR NPs and MRSA was observed based on the auto-fluorescence of BBR molecules, and also on the ultrastructural characteristics of the bacteria. 10^6^ CFU/mL of MRSA strain was inoculated on nutrient agar for 24 h at 37 °C. After that, 1 mL of BBR NPs (2 mg/mL) solution was sprayed on the colonies of bacteria and incubated for 1 h at room temperature. These colonies were then collected and fixed in 2.5% glutaraldehyde/cacodylate for 1 h at room temperature. The sample was washed three times with bi-distilled water and centrifuged at 3.000 rpm for 5 min to remove free BBR NPs. The sediment of samples was divided into two parts: One part was checked under the fluorescence stereomicroscope (M165 FC, Leica), another was used for ultrathin sectioning for TEM observation. The procedure is described in detail in our previous paper [45].

All experimental items were sterilized at 121 °C for 15 min, and operations were carried out in a biosafety cabinet level 2 (Hankook).

#### Statistical analysis

Antimicrobial data were statistically analyzed using one-way analysis of variance (ANOVA) for statistical significance of three groups (*p* < 0.05). Error bars represent one standard deviation.

## Supporting Information

Supporting Information features the raw DLS measurement data.

File 1 Raw DLS data.
