# Supplementary material for: Antibacterial activity of a berberine nanoformulation
Source: Beilstein J Nanotechnol. 2022 Jul 11;13:641–52. doi: 10.3762/bjnano.13.56 (PMC9296985; doi:10.3762/bjnano.13.56)

BEILSTEIN JOURNAL OF NANOTECHNOLOGY

Supporting Information

for

Antibacterial activity of a berberine nanoformulation

Hue Thi Nguyen, Tuyet Nhung Pham, Anh-Tuan Le, Nguyen Thanh Thuy, Tran Quang Huy and Thuy Thi Thu Nguyen

Beilstein J. Nanotechnol. **2022,** *13,* 641–652. doi:10.3762/bjnano.13.56

Raw DLS data

License and Terms: This is a supporting information file under the terms of the Creative Commons Attribution License (https://creativecommons.org/licenses/by/4.0). Please note that the reuse, redistribution and reproduction in particular requires that the author(s) and source are credited and that individual graphics may be subject to special legal provisions.
The license is subject to the *Beilstein Journal of Nanotechnology* terms and conditions: (https://www.beilstein-journals.org/bjnano/terms)

Size Distribution Report by Intensity

v2.1

Malvern

Sample Details

**Sample Name:**	Mau 2 1
**SOP Name:**	Size.sop
**General Notes:**	

**File Name:**	17621.dts	**Dispersant Name:**	Water
**Record Number:**	11	**Dispersant RI:**	1.330
**Material RI:**	1.00	**Viscosity (mPa.s):**	0.8872
**Material Absorbtion:**	0.00	**Measurement Date and Time:**	Thursday, June 17, 2021 5:2...

System

**Temperature (°C):**	25.0	**Duration Used (s):**	60
**Count Rate (kcps):**	257.1	**Measurement Position (mm):**	3.00
**Cell Description:**	Disposable micro cuvette (40µl)	**Attenuator:**	11

Results

			Size (d.nm):	% Intensity	Width (d.nm):
**Z-Average (d.nm):**	530.6	**Peak 1:**	156.0	100.0	16.91
**PdI:**	0.555	**Peak 2:**	0.000	0.0	0.000
**Intercept:**	0.737	**Peak 3:**	0.000	0.0	0.000

Result quality : **Refer to quality report**

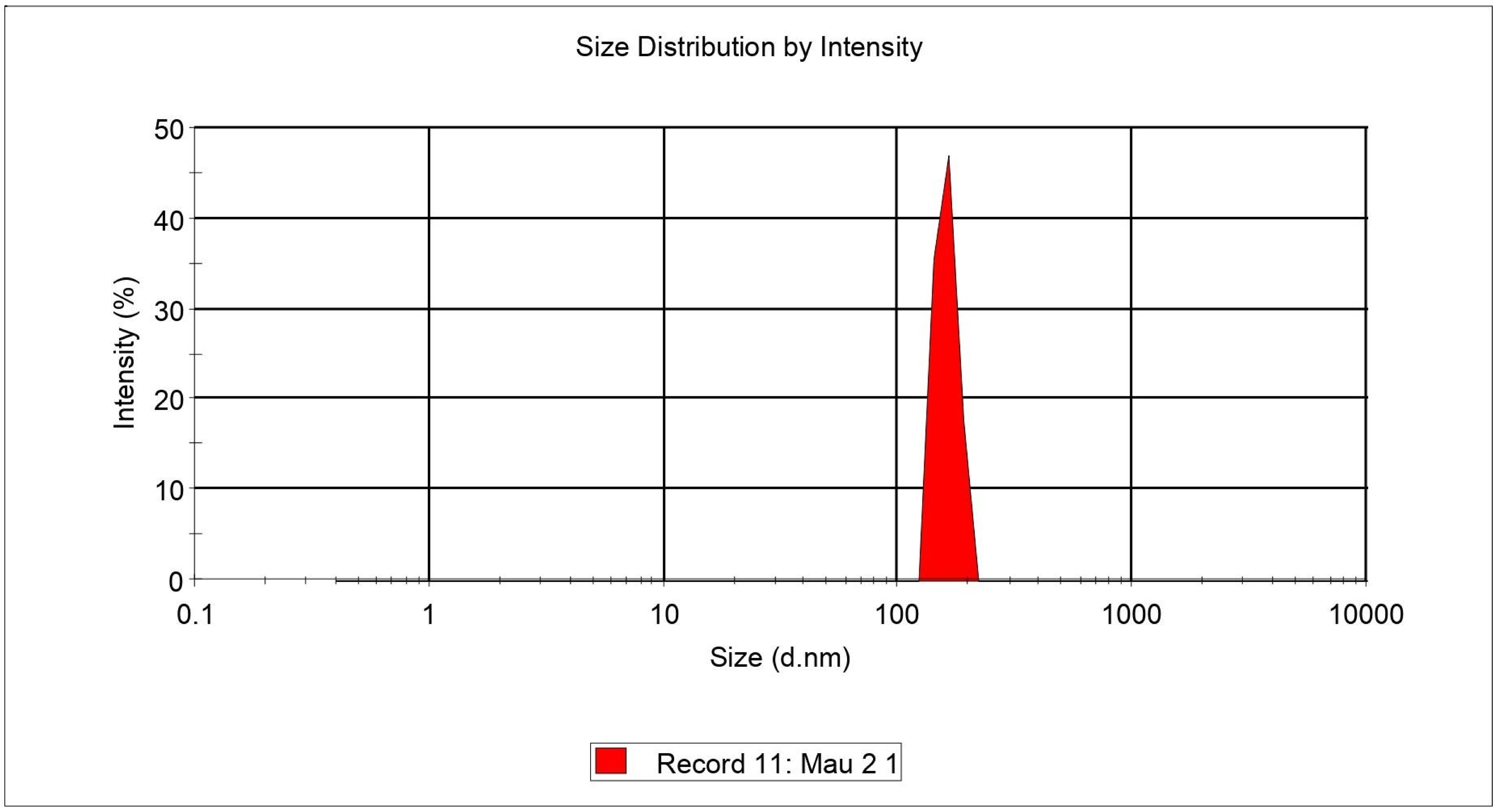

Supplement: File 1 — Raw DLS data. [file Beilstein_J_Nanotechnol-13-641-s001.pdf]
